# From Altered Metabolic and Anthropometric Parameters to Metabolic Syndrome: A Cross-Sectional Survey on the Effectiveness and Safety of Neo-Policaptil^®^ Gel Retard

**DOI:** 10.3390/healthcare13243293

**Published:** 2025-12-15

**Authors:** Elena Gabriele, Roberto Cioeta, Paola Muti, Marta Rigoni, Roberta La Salvia, Andrea Cossu, Emiliano Giovagnoni

**Affiliations:** 1 Aboca SpA, 52037 Sansepolcro, Italy; 2 Department of Biomedical, Surgical and Dental Sciences, University of Milan, 20122 Milan, Italy; 3 Istituto di Ricovero e Cura a Carattere Scientifico I.R.C.C.S. Multimedica, 20099 Milan, Italy

**Keywords:** altered metabolic parameters, metabolic syndrome, real-word evidence, RWE, natural substance-based medical device, safety, effectiveness

## Abstract

**Background/Objectives**: Metabolic syndrome (MetS) and even closely related metabolic and anthropometric alterations require timely intervention to reduce associated risks. Neo-Policaptil^®^ Gel Retard has shown potential in managing both established MetS and early imbalances. To further characterize its real-world impact, a large post-marketing survey was conducted to assess perceived effectiveness, safety, quality of life (QoL), and patterns of use. **Methods**: Between December 2021 and May 2025, patients/child caregivers, pharmacists, and physicians completed online questionnaires via a dedicated web platform. Patients reported their direct experience, while healthcare professionals (HCPs) provided observations on patient use. **Results**: The survey included 2204 patients, 57 child caregivers, 455 physicians, and 387 pharmacists. Overall, 97.7% of patients reported an improvement in their condition. Most physicians (94.3%) and pharmacists (89.1%) rated the overall effectiveness of the product as “*good*” or “*excellent*”. The safety profile was judged “*good*” or “*excellent*” by 84.4% of patients and by over 93% of HCPs. The product was mainly used as monotherapy or in combination with dietary measures and/or physical exercise. **Conclusions**: These findings underscore the product’s effectiveness, safety, tolerability, and positive influence on QoL in both adults and children affected by MetS or by related metabolic and anthropometric imbalances.

## 1. Introduction

Metabolic syndrome (MetS) is a cluster of cardiometabolic risk factors driven by insulin resistance that arises from underlying metabolic dysregulation and predisposes to complications such as diabetes mellitus, elevated uric acid levels, and fatty liver disease [1,2,3,4,5,6,7,8]. The prevalence of MetS among adults was estimated at 35% in the United States during 2011–2016 [9], in parallel with a global doubling of obesity in more than 70 countries and a steeper increase among overweight and obese children [10,11,12,13,14,15]. This trend is largely driven by lifestyle factors [16,17] and contributes to a growing burden of obesity, MetS, and Type 2 Diabetes Mellitus (T2DM), with substantial economic consequences for healthcare systems [18,19,20,21,22].

Even in the absence of a formal MetS diagnosis, single metabolic and anthropometric abnormalities independently increase cardiometabolic risk: increased waist circumference (WC), postprandial hyperglycaemia, prediabetes, and dyslipidaemia are all associated with a higher likelihood of T2DM and cardiovascular disease, largely through visceral adiposity, chronic inflammation, and atherogenic lipoprotein accumulation [23,24,25,26,27]. In pediatric populations, the lack of standardized cut-offs and reliance on age- and sex-specific percentiles make these metabolic alterations difficult to identify [28]. Nonetheless, they are increasingly observed in children and often persist into adulthood, so targeting these abnormalities—whether isolated or combined—is crucial to reducing long-term cardiovascular and liver-related morbidity and mortality [29].

No pharmacologic agents are currently approved specifically for MetS as a unified clinical entity; management instead targets individual components or commonly associated metabolic and cardiovascular (CV) alterations/risk factors [30]. Lifestyle modification, including dietary changes and increased physical activity, is the cornerstone of treatment, especially in children, and has been associated with reductions in MetS and T2DM incidence [31,32,33]. However, long-term adherence is frequently suboptimal, contributing to the persistent high prevalence of these conditions [6,34]. Pharmacological therapies (e.g., statins and fibrates for dyslipidaemia, insulin sensitisers such as metformin, and anti-obesity agents including orlistat and Glucagon-Like Peptide-1 [GLP-1] analogues) are often used as adjuncts, but raise concerns regarding adverse events, limited long-term safety data, and, in children, the appropriateness of early pharmacological exposure [35,36,37,38,39,40,41]. For GLP-1 analogues, in particular, reimbursement for weight management is not allowed in most countries, creating major affordability issues due to their high cost. This scenario underscores the need for non-pharmacological interventions with a favourable safety profile and minimal side effects, suitable for long-term use alongside healthy lifestyle measures.

Metarecod (Aboca S.p.a.—Sansepolcro, Arezzo—Italy) [42] is a natural, biodegradable substance-based medical device (SBMD) containing the vegetal complex Neo-Policaptil^®^ Gel Retard (NPGR), indicated for the management of one or more altered metabolic and anthropometric parameters, including blood cholesterol, triglycerides, blood glucose and WC, up to MetS. When alterations occur individually or in combination, NPGR exerts targeted effects on the altered parameter(s) while supporting overall metabolic regulation. Clinical studies have shown that this SBMD can improve lipid and glucose profiles and reduce WC, in line with the known effects of water-holding dietary fibres [43,44,45,46,47,48]. Unlike conventional dietary fibres [49,50], the patented formulation [51] forms a large and viscous gel in the intestine, more than its individual ingredients alone. This enhances sequestration of dietary lipids and carbohydrates, enabling a clinically relevant, non-pharmacological modulation of key metabolic parameters, as demonstrated in controlled studies [43,44,47]. A systematic review by Guarino et al. summarized data from obese children and adolescents and from overweight or obese adults, with or without MetS and/or T2DM, further supporting its effectiveness and safety [44].

However, most available evidence on NPGR derives from controlled trials, while real-world data on its effectiveness and safety remain limited. In this context, the present article presents the results of a post-marketing clinical follow-up survey designed to evaluate the effectiveness, tolerability, safety, and usage patterns across different user groups (patients/child caregivers, pharmacists and physicians), in compliance with the Regulation (EU) 2017/745 [52].

## 2. Materials and Methods

### 2.1. Product

NPGR is a complex of different types of dietary fibre based on polysaccharide macromolecules from *Avena sativa* L., *Opuntia ficus-indica* L., *Amorphophallus konjac K. Koch, Linum usatissimum* L., *Althea officinalis* L., *Tilia platyphyllos Scop.*, and *Cichorium intybus* L.; other ingredients include powdered stevia leaves, arabic gum, natural orange, and peach flavouring.

The product acts locally in the intestine by forming a viscous gel that modifies the physical characteristics of the intestinal contents, slowing and reducing the absorption of carbohydrates and lipids (retard effect). This mechanism supports glycaemic control, appetite regulation, and cholesterol balance through reduced bile acid reabsorption, rebalancing the intestinal flora and normalizing bowel transit. This formulation is suitable for adults and for children from 8 years of age, and its use should be combined with a balanced diet and regular physical activity [42,51].

### 2.2. Survey Design

The present survey reports RWD collected between December 2021 and May 2025, spontaneously provided by three user groups: patients and child caregivers (including adults using the device themselves and caregivers reporting on its use in children aged 8 to over 12 years), physicians (both general practitioners and specialists), and pharmacists. This is an ongoing process in which all RWD are collected via Web Framework Page on a GxP-validated platform (version 2.3.1.2) [53], which allows users to complete an online survey based on specific questionnaires. The platform is fully compliant with GDPR requirements, ensuring data protection and regulatory compliance. All data were collected and analyzed in anonymized form. Users could access the surveys through the patients or HCP section of the manufacturer’s website.

Product use was verified by entering both the batch number and unique code printed on the device packaging at the beginning of the online questionnaire. Patients/child caregivers were asked about their personal experience associated with the device, while pharmacists and physicians were requested to describe patients’ experience based on their own direct observation, thereby providing an indirect validation of patient-reported data. The questionnaires, specifically tailored to each responder category, were developed and tested for repeatability [54] through scientific collaboration with the University of Milan.

The survey focused mainly on perceived effectiveness of the product, satisfaction with product use, safety and tolerability, compliance with the recommended dosage, QoL after treatment, concomitant treatments, conditions for which the product was used/prescribed, improvement and timing of problem resolution and clarity of device information.

Answers regarding QoL improvement, safety/tolerability and effectiveness were assessed using a single-item question with a 5-point Likert scale: ‘*not at all/poor*’, ‘*somewhat/adequate*’, ‘*moderately/fair*’, ‘*greatly/good*’, ‘*extremely/excellent*’. The survey collected qualitative data, summarized as percentage distributions of responses. The questionnaires for patients, physicians, and pharmacists are available in the Supplementary Materials. At the end of the questionnaire, respondents could report any adverse event or suspected interaction with the product to the Medical Vigilance Department via a dedicated safety mailbox.

### 2.3. Statistical Analysis

The sample size for the survey was determined on the basis of the patient questionnaire, focusing on items assessing satisfaction with safety and tolerability, QoL, and symptom improvement. Assuming a population size of 20,000 or more, a 2% margin of error, a 95% confidence level, and a conservative response rate of 50%, the required sample size was estimated using an online tool (http://www.raosoft.com/samplesize.html, accessed on 1 May 2025) [55]), yielding a target of 2144 respondents.

Descriptive analyses were performed for each question, reporting both absolute frequencies and response percentages. For single- and multiple-choice items, the percentage for each answer represents the proportion of responders who selected that option.

## 3. Results

### 3.1. Patients/Child Caregivers

A total of 2261 patients/child caregivers participated in the survey. The most represented age ranges were 51–64 years (49.7%, N = 1124) and 31–50 years (31.5%, N = 713). The most frequently reported sources of information were recommendations from pharmacists (22.8%, N = 516) or doctors (18.6%, N = 421), and the Internet (16.3%, N = 369). A very high rate of direct use was observed: 2204 (97.5%) patients used the product personally, while 57 (3.5%) reported administering it to a child.

#### 3.1.1. Direct Use

**Figure 1:**
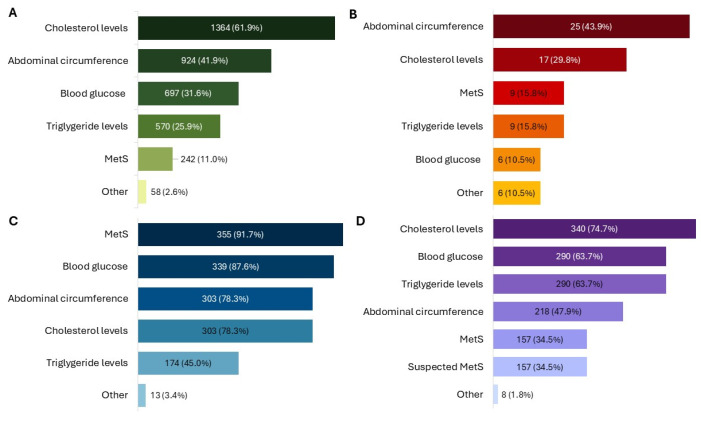
Conditions for which the product was used or prescribed, as reported by patients (**A**), child caregivers (**B**), physicians (**C**), and pharmacists (**D**). All results in the figure refer to multiple-response questions, with the percentages indicating the proportion of responders who selected each response. MetS: Metabolic Syndrome.

A clear majority of patients (62.8%, N = 1385) reported a tendency toward self-medication, and product use was primarily associated with the management of cholesterol levels (61.9%, N = 1364) (Figure 1A).

**Figure 2:**
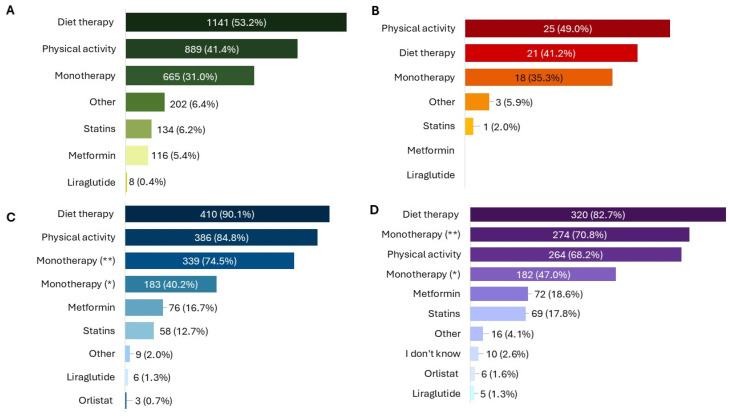
Association of NPGR with other therapeutic interventions, as reported by patients (**A**), child caregivers (**B**), physicians (**C**), and pharmacists (**D**). All results in the figure refer to multiple-response questions, with the percentages indicating the proportion of responders who selected each response. * = previous unsuccessful therapies; ** = no previous therapies.

In 11.0% (N = 242) of cases, the product was used following a medical diagnosis of MetS. Among these, the most frequent altered parameters in addition to high WC were the levels of cholesterol (64.9%, N = 157) and blood sugar (57.0%, N = 138). The most common treatment durations were 3 months (28.7%, N = 615), less than 1 month (23.5%, N = 504) and 2 months (21.5%, N = 462). Additionally, 941 patients (43.9%) reported using the product for the first time. Patients reported using the product as the only therapy in 31.0% (N = 665) of cases, while many combined it with diet therapy (53.1%, N = 1141) or physical activity (41.4%, N = 889) (Figure 2A).

**Figure 3:**
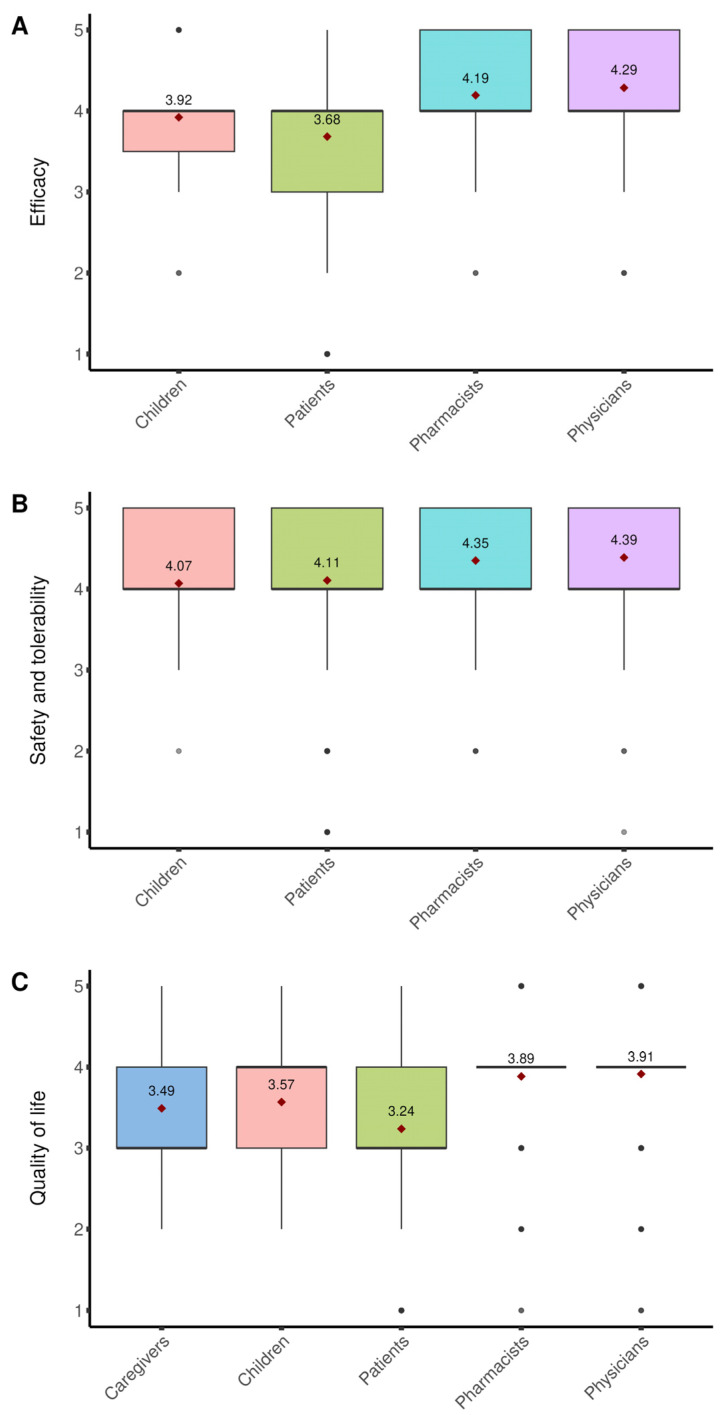
Effectiveness, safety/tolerability and impact on QoL. Boxplots represent Likert scale ratings for (**A**) perceived efficacy, (**B**) safety and tolerability, and (**C**) impact on quality of life (QoL) of NPGR, as reported by different groups of participants (patient/child caregivers, pharmacists, physicians). For each item, responses were transformed into numerical values from 5 (most favourable) to 1 (least favourable). The boxplots show the distribution of ratings within each group, with the mean value indicated by a red diamond. All data refer to single-response questions.

**Figure 4:**
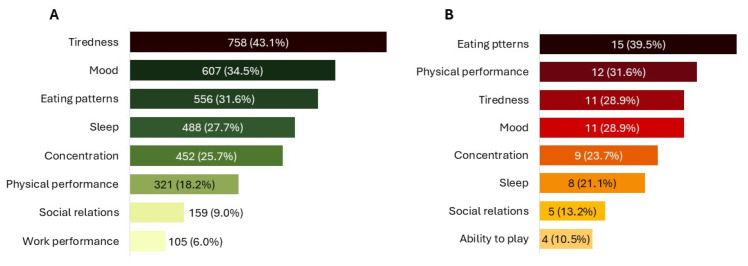
Specific impact on QoL. (**A**) QoL improvements experienced by patients. (**B**) QoL improvements in children, as reported by caregivers. All data refer to multiple-response questions, with the percentages indicating the proportion of responders who selected each response.

Use of NPGR improved patients’ condition in 97.7% (N = 2097) of cases, with most describing the improvement as “*moderate*” (45.3%, N = 971) or “*great*” (29.8%, N = 639) (Figure 3A). Overall, 65.1% (N = 1397) of patients rated their satisfaction with the product as “*good*” or “*excellent*”. In terms of QoL, 79.7% (N = 1711) of patients reported at least a moderate improvement (Figure 3C). Around or over one third reported reduced tiredness (43.1%, N = 758), improved mood (34.5%, N = 607), and greater freedom in eating (31.6%, N = 556) (Figure 4A). The first beneficial effects were reported within 3 months in 76.3% (N = 1638) of cases, including 24.5% (N = 526) who noticed improvements within the firstmonth.

Most patients (76.0%, N = 1631) took the recommended two sachets per day, while 23.6% (N = 506) used half the dose. The product was usually taken before the two main meals (97.0%, N = 2082) and followed by an additional half glass of water (92.1%, N = 1976). Among the 1313 patients taking other medications, 81.3% (N = 1067) reported taking them two hours apart from NPGR.

#### 3.1.2. Child Administration

Children receiving the product were almost evenly distributed by sex (52.6% female, N = 30), with all age ranges from 8 to over 12 years represented and 40.0% (N = 23) aged over 12 years. Caregivers mainly consulted a doctor for their children’s symptoms (59.7%, N = 34), and use of the product was most often linked to specific altered parameters, particularly increased WC (43.9%, N = 25) (Figure 1B). The product was administered as monotherapy in 35.3% (N = 18) of children, while almost half also followed a physical activity plan (49.0%, N = 25) or a diet therapy (41.2%, N = 21) (Figure 2B).

Caregivers reported an improvement in the child’s condition, with 49.0% (N = 25) rating it as “*good*” or “*excellent*” (Figure 3A), and overall satisfaction with the product was “*good*” or “*excellent*” in 74.5% (N = 38). All caregivers observed an improvement in the child’s QoL, described as “*great*” or “*extreme*” in 56.9% (N = 29) of cases (Figure 3C), with the greatest benefits reported for eating patterns (39.5%, N = 15) and physical activity (31.6%, N = 12) (Figure 4B). Caregivers also reported an improvement in their own QoL, most frequently described as “*moderate*” (37.3%, N = 19) or “*great*” (35.3%, N = 18) (Figure 3C).

Most children (66.7%, N = 34) received two sachets per day, while 31.4% (N = 16) received one per day. The product was usually administered before the two main meals (94.1%, N = 48 and followed by an additional half glass of water (84.3%, N = 43). Among the 20 children taking other medications, 70.0% (N = 14) were given NPGR two hours apart. The first beneficial effects were reported within 3 months in 76.4% (N = 39) of cases, with 23.5% (N = 12) noticing improvements within less than one month.

The safety and tolerability of the product were rated as “*good*” or “*excellent*” by 84.4% (N = 1908) of patients/child caregivers (Figure 3B). Across the entire survey, only 11 patients reported non-serious undesirable side effects (USEs) considered at least possibly related to the use of the product, mainly involving the gastrointestinal system. Communication clarity (Indications, Instructions for use, and Warnings) was judged “*good*” or “e*xcellent*” by over 93% of patients.

### 3.2. Physicians

Among the 455 physicians, the largest groups included pediatricians (28.6%, N = 130), GPs (22.6%, N = 103), and nutritionists (21.3%, N = 97). The age ranges most commonly reported for their patients were 31–50 (62.2%, N = 283), 51–64 (54.3%, N = 247), and 18–30 (32.3%, N = 147) years. The product was mainly prescribed for MetS (78.0%, N = 355), high blood sugar (74.5%, N = 339), elevated LDL cholesterol (66.6%, N = 303), and increased WC (66.6%, N = 303) (Figure 1C).

Treatment was usually prescribed for 3 months (75.4%, N = 343), followed by 2 months (29.0%, N = 132) and one month (8.4%, N = 38). Almost all physicians (99.8%, N = 454) recommended administration before the two main meals, and 91.0% (N = 414) advised taking it two hours apart from other medications. The product was prescribed as monotherapy mainly in patients naïve to other treatments (74.5%, N = 339), but also in those who had failed previous therapies (40.2%, N = 183). NPGR was associated with diet in 90.1% (N = 410) of cases and with physical activity in 84.8% (N = 386) (Figure 2C).

Overall effectiveness was rated as “*good*” by 59.3% (N = 270) and “*excellent*” by 35.0% (N = 159) (Figure 3A). Satisfaction was highest for effects on blood glucose (50.3% [N = 229] “*greatly*”, 23.1% [N = 105] “*extremely*”), followed by MetS (48.4% [N = 220] “*greatly*”, 16.0% [N = 73] “*extremely*”), LDL cholesterol (49.5% [N = 225] “*greatly*”, 11.7% [N = 53] “*extremely*”), and WC (46.4% [N = 211] “*greatly*”, 13.0% [N = 59] “*extremely*”). Regarding QoL, 65.1% (N = 296) of physicians rated the impact as “*good*”. Most physicians (82.0%, N = 418) reported that patients were likely to repeat treatment after the first cycle, and the first improvements were generally observed within 1–3 months (42.0% [N = 191] in 2 months, 41.3% [N = 188] in one month, 35.8% [N = 163] in 3 months), while only 3 physicians (0.7%) reported no improvements.

The product was consistently rated highly for safety and tolerability, with 48.4% (N = 220) of physicians rating it as “*good*” and 45.7% (N = 208) as “*excellent*” (Figure 3B). Across the survey, only 4 physicians reported non-serious USEs at least possibly related to the product, mainly gastrointestinal (one case of skin rash), and interactions with other treatments were noted by only 0.66% of physicians. Communication was judged very favourably: over 98% rated the information on the box and leaflet (Instructions, Warnings, Indications) as *“good*” or “*excellent*”, and the quality of scientific information from the company was rated as “*excellent*” by 73.9% (N = 336) and “*good*” by 25.3% (N = 115).

### 3.3. Pharmacists

Among 387 pharmacists who participated, all reported receiving feedback from their customers. Most dispensed the product through independent private pharmacies (50.4%, N = 195). Treatment duration was mainly 3 months (60.5%, N = 234) or 2 months (47.3%, N = 183). The product was primarily recommended for high cholesterol levels (87.9%, N = 340), triglycerides (74.9%, N = 290), and blood sugar (74.9%, N = 290) (Figure 1D). Almost all pharmacists recommend taking NPGR before the two main meals (99.4%, N = 383) and two hours apart from other medications (97.4%, N = 377). The product was most often used in association with diet therapy (82.7%, N = 320) and physical activity (68.2%, N = 264), but also as monotherapy (70.8%, N = 274) (Figure 2D).

Most pharmacists rated overall perceived effectiveness as “*good”* (58.4%, N = 226) or “*excellent*” (30.8%, N = 119) (Figure 3A). Customer satisfaction with improvements in individual parameters was highest for blood sugar (46.8% [N = 181] “*greatly*”, 11.4% [N = 44] “*extremely*”), cholesterol (46.5% [N = 180] “*greatly*”, 12.9% [N = 50] “*extremely*”), and WC (33.1% [N = 128] “*greatly*”, 9.3% [N = 36] “*extremely*”). Improvement in QoL was mostly rated at least “*good*” (67.7%, N = 262). Most pharmacists (79.6%, N = 371) reported that customers tend to repeat treatment after a first cycle, and feedback of clinical improvement was mainly received within 2 months (48.6%, N = 188) or one month (39.0%, N = 151), with only 3 pharmacists (0.8%) reporting no improvement.

Safety and tolerability were rated as “*good*” by 50.7% (N = 196) of pharmacists and “*excellent*” by 42.6% (N = 165) (Figure 3B). No USEs associated with device use were reported, and only 1.03% mentioned interactions with concomitant treatments. Communication clarity (Instructions, Warnings, Indications) was rated “*good*” or “*excellent*” by over 96%, and the quality of scientific communication from the company was judged “*excellent*” by 50.1% (N = 194) and “*good*” by 45.5% (N = 176) of pharmacists.

## 4. Discussion

This research provides real-world evidence (RWE) from a large post-marketing survey involving patients/child caregivers, physicians, and pharmacists, evaluating the perceived effectiveness, tolerability, safety and usage patterns of NPGR. As commonly observed in real-world practice, it is often challenging to accurately assess these outcomes [56,57]. In this context, RWE surveys offer complementary insights on patient outcomes in routine practice, broadening the perspective beyond clinical trials [53,58].

All direct indicators of effectiveness (e.g., symptomatic improvement, speed of symptom resolution) and indirect indicators (e.g., QoL improvement) showed high levels of appreciation for product performance among patients, supporting the positive evidence reported in previous studies on the device [43,44,45,46,47,48]. The high safety ratings reported by patients suggest a favourable benefit-risk profile, which likely contributed to overall satisfaction.

Although RWD on children were limited, all indicators of effectiveness were also confirmed in this user group. In the pediatric sample, the product was almost never used in combination with pharmacological treatment, reflecting a preference for interventions with a lower physiological burden. This is particularly relevant given the limited number of medications approved for pediatric use [39]. Child caregivers expressed high satisfaction, in line with preferences for safe and well-tolerated interventions. These findings are consistent with earlier clinical studies, such as those by Stagi et al. [46], which showed that use of the product in obese children and adolescents with type 1 diabetes and MetS led to significant improvements in BMI, waist circumference (WC), glycaemic control, and insulin requirements after just three months of treatment.

Both pharmacists and physicians confirmed effectiveness, safety and tolerability in clinical practice, basing their assessments on both patient feedback and direct observation. They also reported high satisfaction with its effects on individual risk factors and on MetS overall.

Fibres play a key role in modulating metabolic parameters: soluble fibres like glucomannan and pectin lower LDL cholesterol [59], while others reduce postprandial glucose peaks [60,61]; high-fibre diets are linked to reduced risk of MetS through combined effects on weight loss, glycaemic control, and lipid profile [62,63,64].

Accordingly, the effectiveness of NPGR is attributed to its water-holding fibres, which form viscous gels in the intestine [51,65,66], slowing nutrient absorption and supporting gut function [42]. Several fibre-based therapies (e.g., glucomannan, inulin, psyllium) have shown benefits on metabolic parameters, but with variable efficacy and tolerability [59,60,61,62,63,64]. NPGR’s unique gel-forming mechanism may enhance appetite regulation [48], and recent studies have shown non-inferiority to metformin for glycemic control and better lipid-lowering and tolerability [43,67].

Previous research indicates that, combined with a low-glycaemic index diet, the product can reduce BMI and metabolic risk factors in obese pediatric patients [58], and that can be effects on satiety and appetite-regulating hormones, likely mediated by delayed nutrient absorption and stimulation of GLP-1 secretion [48].

Consistent with these findings, in the present survey, many patients used NPGR with diet therapy, and high satisfaction with effects on WC suggests support for dietary adherence.

Notably, in a recent single-blind randomized trial [67], the product was non-inferior to metformin (MTF) in obese adults with MetS and coexisting T2DM, while providing better lipid-lowering effects and tolerability; improvements in glucose, insulin, and lipid metabolism appeared within 3 months, in line with the timing of first benefits reported in the present survey, and persisted up to 6 months, suggesting a sustained effect on intestinal absorption. Evidence from animal models further indicates that NPGR may beneficially modulate the gut microbiota [65], consistent with broader data showing that dietary fibres and carbohydrate polymers shape microbial composition and promote the production of health-supporting metabolites [68]. Unlike MTF, NPGR may therefore deliver multiple metabolic benefits with the favourable safety profile of a device based on natural molecules, and in this survey, safety and tolerability emerged as major reasons for its use, with consistently positive ratings across all user groups and minimal reported interactions.

These RWD complement randomized trial data, extending evidence to real-world settings and showing consistent benefits across target groups. These findings further enrich existing clinical trial evidence by providing additional real-world insights.

Beyond clinical outcomes, the product has demonstrated biodegradability under OECD 301F conditions, with substantial transformation within 28 days, and metabolomic profiling has confirmed the transformation of selected compounds, reinforcing its eco-compatibility [69].

A limitation of this work is the relative under-representation of age groups younger than 30 years in the patient sample, which may introduce some bias in the interpretation of questionnaire responses. However, information on younger age groups was partly captured through pharmacists’ and physicians’ responses, allowing reliable conclusions to be drawn for the overall population. A slight propensity for self-medication was observed, but this did not affect consistency between patient and HCP reports.

## 5. Conclusions

The results of this post-marketing survey reinforce the evidence supporting NPGR’s effectiveness, safety, and high user satisfaction, as consistently reported by patients/child caregivers, pharmacists, and physicians across all age groups represented. Together with the effectiveness and safety data from existing randomized controlled trials, these findings support the Neo-Policaptil^®^ Gel Retard complex as an effective and safe option for the treatment of altered metabolic parameters and MetS in children, adolescents, and adults. As a non-pharmacological intervention, the product offers a favourable risk–benefit profile for the clinical management of significant metabolic and anthropometric disturbances.

## Data Availability

The data presented in this study are available on request from the corresponding author. The data are not publicly available due to privacy and ethical restrictions.

## Supplementary Materials

The following supporting information can be downloaded at: https://www.mdpi.com/article/10.3390/healthcare13243293/s1.

## Abbreviations

The following abbreviations are used in this manuscript:

MetS	Metabolic syndrome
QoL	Quality of life
HCPs	Healthcare professionals
TG	Triglycerides
HDL-C	High-density lipoprotein cholesterol
T2DM	Type 2 diabetes mellitus
BMI	Body mass index
IFG	Impaired fasting glucose
IGT	Impaired glucose tolerance
MTF	Metformin
SBMD	Substance-based medical device
NPGR	Neo-Policaptil® Gel Retard
RWD	Real world data
PGR	Policaptil Gel Retard®
GPs	General practitioners
GLP-1	Glucagon-like peptide-1
USEs	Unusual serious events
RWE	Real-world evidence
MAFLD	Metabolic non-alcoholic fatty liver disease

## References

[1] Alberti K.G.M.M., Eckel R.H., Grundy S.M., Zimmet P.Z., Cleeman J.I., Donato K.A., Fruchart J.-C., James W.P.T., Loria C.M., Smith S.C. (2009). Harmonizing the Metabolic Syndrome: A Joint Interim Statement of the International Diabetes Federation Task Force on Epidemiology and Prevention; National Heart, Lung, and Blood Institute; American Heart Association; World Heart Federation; International Atherosclerosis Society; and International Association for the Study of Obesity. Circulation.

[2] Zimmet P., Alberti K.G.M., Kaufman F., Tajima N., Silink M., Arslanian S., Wong G., Bennett P., Shaw J., Caprio S. (2007). The Metabolic Syndrome in Children and Adolescents? An IDF Consensus Report. Pediatr. Diabetes.

[3] Nsiah K., Shang V.O., Boateng K., Mensah F. (2015). Prevalence of Metabolic Syndrome in Type 2 Diabetes Mellitus Patients. Int. J. Appl. Basic Med. Res..

[4] James M., Varghese T.P., Sharma R., Chand S. (2020). Association Between Metabolic Syndrome and Diabetes Mellitus According to International Diabetic Federation and National Cholesterol Education Program Adult Treatment Panel III Criteria: A Cross-Sectional Study. J. Diabetes Metab. Disord..

[5] Burgert T.S., Taksali S.E., Dziura J., Goodman T.R., Yeckel C.W., Papademetris X., Constable R.T., Weiss R., Tamborlane W.V., Savoye M. (2006). Alanine Aminotransferase Levels and Fatty Liver in Childhood Obesity: Associations with Insulin Resistance, Adiponectin, and Visceral Fat. J. Clin. Endocrinol. Metab..

[6] DeBoer M.D. (2019). Assessing and Managing the Metabolic Syndrome in Children and Adolescents. Nutrients.

[7] Morrison J.A., Friedman L.A., Wang P., Glueck C.J. (2008). Metabolic Syndrome in Childhood Predicts Adult Metabolic Syndrome and Type 2 Diabetes Mellitus 25 to 30 Years Later. J. Pediatr..

[8] Muntner P., Menke A., Srinivasan S., Patel D.A., Chen W., Berenson G. (2008). Impact of Childhood Metabolic Syndrome Components on the Risk of Elevated Uric Acid in Adulthood: The Bogalusa Heart Study. Am. J. Med. Sci..

[9] Hirode G., Wong R.J. (2020). Trends in the Prevalence of Metabolic Syndrome in the United States, 2011–2016. JAMA.

[10] Forouzanfar M.H., Afshin A., Alexander L.T., Anderson H.R., Bhutta Z.A., Biryukov S., Brauer M., Burnett R., Cercy K., Charlson F.J. (2016). Global, Regional, and National Comparative Risk Assessment of 79 Behavioural, Environmental and Occupational, and Metabolic Risks or Clusters of Risks, 1990–2015: A Systematic Analysis for the Global Burden of Disease Study 2015. Lancet.

[11] (2017). The GBD 2015 Obesity Collaborators. Health Effects of Overweight and Obesity in 195 Countries over 25 Years. N. Engl. J. Med..

[12] Gepstein V., Weiss R. (2019). Obesity as the Main Risk Factor for Metabolic Syndrome in Children. Front. Endocrinol..

[13] Swinburn B.A., Kraak V.I., Allender S., Atkins V.J., Baker P.I., Bogard J.R., Brinsden H., Calvillo A., De Schutter O., Devarajan R. (2019). The Global Syndemic of Obesity, Undernutrition, and Climate Change: The Lancet Commission Report. Lancet.

[14] Reinehr T., de Sousa G., Toschke A.M., Andler W. (2007). Comparison of Metabolic Syndrome Prevalence Using Eight Different Definitions: A Critical Approach. Arch. Dis. Child..

[15] Pelin A.-M., Mătăsaru S. (2012). Metabolic Syndrome in Obese Children and Adolescents. Rev. Med. Chir. Soc. Med. Nat. Iasi.

[16] Taskinen M.-R., Packard C.J., Borén J. (2019). Dietary Fructose and the Metabolic Syndrome. Nutrients.

[17] Mortera R.R., Bains Y., Gugliucci A. (2019). Fructose at the Crossroads of the Metabolic Syndrome and Obesity Epidemics. Front. Biosci. (Landmark Ed.).

[18] Bhupathiraju S.N., Hu F.B. (2016). Epidemiology of Obesity and Diabetes and Their Cardiovascular Complications. Circ. Res..

[19] Vaquero Alvarez M., Aparicio-Martinez P., Fonseca Pozo F.J., Valle Alonso J., Blancas Sánchez I.M., Romero-Saldaña M. (2020). A Sustainable Approach to the Metabolic Syndrome in Children and Its Economic Burden. Int. J. Environ. Res. Public Health.

[20] Mancia G., Bombelli M., Facchetti R., Casati A., Ronchi I., Quarti-Trevano F., Arenare F., Grassi G., Sega R. (2010). Impact of Different Definitions of the Metabolic Syndrome on the Prevalence of Organ Damage, Cardiometabolic Risk and Cardiovascular Events. J. Hypertens..

[21] Steinberger J., Daniels S.R., Eckel R.H., Hayman L., Lustig R.H., McCrindle B., Mietus-Snyder M.L. (2009). Progress and Challenges in Metabolic Syndrome in Children and Adolescents: A Scientific Statement from the American Heart Association Atherosclerosis, Hypertension, and Obesity in the Young Committee of the Council on Cardiovascular Disease in the Young; Council on Cardiovascular Nursing; and Council on Nutrition, Physical Activity, and Metabolism. Circulation.

[22] Yates K.F., Sweat V., Yau P.L., Turchiano M.M., Convit A. (2012). Impact of Metabolic Syndrome on Cognition and Brain: A Selected Review of the Literature. Arterioscler. Thromb. Vasc. Biol..

[23] Lee C.M.Y., Huxley R.R., Wildman R.P., Woodward M. (2008). Indices of Abdominal Obesity Are Better Discriminators of Cardiovascular Risk Factors than BMI: A Meta-Analysis. J. Clin. Epidemiol..

[24] Schneider H.J., Glaesmer H., Klotsche J., Böhler S., Lehnert H., Zeiher A.M., März W., Pittrow D., Stalla G.K., Wittchen H.-U. (2007). Accuracy of Anthropometric Indicators of Obesity to Predict Cardiovascular Risk. J. Clin. Endocrinol. Metab..

[25] Arumugam V., Lee J.-S., Nowak J.K., Pohle R.J., Nyrop J.E., Leddy J.J., Pelkman C.L. (2008). A High-Glycemic Meal Pattern Elicited Increased Subjective Appetite Sensations in Overweight and Obese Women. Appetite.

[26] Tabák A.G., Herder C., Rathmann W., Brunner E.J., Kivimäki M. (2012). Prediabetes: A High-Risk State for Diabetes Development. Lancet.

[27] Ference B.A., Ginsberg H.N., Graham I., Ray K.K., Packard C.J., Bruckert E., Hegele R.A., Krauss R.M., Raal F.J., Schunkert H. (2017). Low-Density Lipoproteins Cause Atherosclerotic Cardiovascular Disease. 1. Evidence from Genetic, Epidemiologic, and Clinical Studies. A Consensus Statement from the European Atherosclerosis Society Consensus Panel. Eur. Heart J..

[28] De Onis M. (2007). Development of a WHO Growth Reference for School-Aged Children and Adolescents. Bull. World Health Organ..

[29] Magnussen C.G., Koskinen J., Chen W., Thomson R., Schmidt M.D., Srinivasan S.R., Kivimäki M., Mattsson N., Kähönen M., Laitinen T. (2010). Pediatric Metabolic Syndrome Predicts Adulthood Metabolic Syndrome, Subclinical Atherosclerosis, and Type 2 Diabetes Mellitus but Is No Better Than Body Mass Index Alone: The Bogalusa Heart Study and the Cardiovascular Risk in Young Finns Study. Circulation.

[30] Mohamed S.M., Shalaby M.A., El-Shiekh R.A., El-Banna H.A., Emam S.R., Bakr A.F. (2023). Metabolic Syndrome: Risk Factors, Diagnosis, Pathogenesis, and Management with Natural Approaches. Food Chem. Adv..

[31] Lee L., Sanders R.A. (2012). Metabolic Syndrome. Pediatr. Rev..

[32] Caranti D.A., de Mello M.T., Prado W.L., Tock L., Siqueira K.O., de Piano A., Lofrano M.C., Cristofalo D.M.J., Lederman H., Tufik S. (2007). Short- and Long-Term Beneficial Effects of a Multidisciplinary Therapy for the Control of Metabolic Syndrome in Obese Adolescents. Metabolism.

[33] Knowler W.C., Barrett-Connor E., Fowler S.E., Hamman R.F., Lachin J.M., Walker E.A., Nathan D.M. (2002). Diabetes Prevention Program Research Group Reduction in the Incidence of Type 2 Diabetes with Lifestyle Intervention or Metformin. N. Engl. J. Med..

[34] Wang L.X., Gurka M.J., Deboer M.D. (2018). Metabolic Syndrome Severity and Lifestyle Factors among Adolescents. Minerva Pediatr..

[35] Rask Larsen J., Dima L., Correll C.U., Manu P. (2018). The Pharmacological Management of Metabolic Syndrome. Expert. Rev. Clin. Pharmacol..

[36] Fornari E., Maffeis C. (2019). Treatment of Metabolic Syndrome in Children. Front. Endocrinol..

[37] Morales-Palomo F., Moreno-Cabañas A., Ramirez-Jimenez M., Alvarez-Jimenez L., Valenzuela P.L., Lucia A., Ortega J.F., Mora-Rodriguez R. (2021). Exercise Reduces Medication for Metabolic Syndrome Management: A 5-Year Follow-Up Study. Med. Sci. Sports Exerc..

[38] Khera R., Murad M.H., Chandar A.K., Dulai P.S., Wang Z., Prokop L.J., Loomba R., Camilleri M., Singh S. (2016). Association of Pharmacological Treatments for Obesity with Weight Loss and Adverse Events: A Systematic Review and Meta-Analysis. JAMA.

[39] Weihe P., Weihrauch-Blüher S. (2019). Metabolic Syndrome in Children and Adolescents: Diagnostic Criteria, Therapeutic Options and Perspectives. Curr. Obes. Rep..

[40] Davidson M.B. (2020). Metformin Should Not Be Used to Treat Prediabetes. Diabetes Care.

[41] Mead E., Atkinson G., Richter B., Metzendorf M.-I., Baur L., Finer N., Corpeleijn E., O’Malley C., Ells L.J. (2016). Drug Interventions for the Treatment of Obesity in Children and Adolescents. Cochrane Database Syst. Rev..

[42] Aboca. https://www.aboca.com/product/metarecod/.

[43] Guarino G., Strollo F., Della-Corte T., Satta E., Romano C., Alfarone C., Corigliano G., Corigliano M., Cozzolino G., Brancario C. (2022). Comparison between Policaptil Gel Retard and Metformin by Testing of Temporal Changes in Patients with Metabolic Syndrome and Type 2 Diabetes. Diabetology.

[44] Guarino G., Strollo F., Malfertheiner P., Della Corte T., Stagi S., Masarone M., Gentile S. (2022). Efficacy and Safety of a Polysaccharide-Based Natural Substance Complex in the Treatment of Obesity and Other Metabolic Syndrome Components: A Systematic Review. Front. Drug Saf. Regul..

[45] Centorame G., Baldassarre M.P.A., Di Dalmazi G., Gambacorta F., Febo F., Consoli A., Formoso G. (2022). Policaptil Gel Retard^®^ Reduces Body Weight and Improves Insulin Sensitivity in Obese Subjects. Ital. J. Food Sci..

[46] Stagi S., Papacciuoli V., Ciofi D., Piccini B., Farello G., Toni S., Ferrari M., Chiarelli F. (2021). Retrospective Evaluation on the Use of a New Polysaccharide Complex in Managing Paediatric Type 1 Diabetes with Metabolic Syndrome (MetS). Nutrients.

[47] Guarino G., Strollo F., Della Corte T., Satta E., Gentile S. (2023). Effect of Neo-Policaptil Gel Retard on Liver Fat Content and Fibrosis in Adults with Metabolic Syndrome and Type 2 Diabetes: A Non-Invasive Approach to MAFLD. Diabetes Ther..

[48] Fornari E., Morandi A., Piona C., Tommasi M., Corradi M., Maffeis C. (2020). Policaptil Gel Retard Intake Reduces Postprandial Triglycerides, Ghrelin and Appetite in Obese Children: A Clinical Trial. Nutrients.

[49] Deehan E.C., Mocanu V., Madsen K.L. (2024). Effects of Dietary Fibre on Metabolic Health and Obesity. Nat. Rev. Gastroenterol. Hepatol..

[50] Papathanasopoulos A., Camilleri M. (2010). Dietary Fiber Supplements: Effects in Obesity and Metabolic Syndrome and Relationship to Gastrointestinal Functions. Gastroenterology.

[51] Mercati V., Marini F., Aboca SpA Societa Agricola (2023). New System with Emerging Properties for Use in the Treatment of Metabolic Syndrome.

[52] European Union (2017). Regulation (EU) 2017/745 of the European Parliament and of the Council of 5 April 2017 on Medical Devices, Amending Directive 2001/83/EC, Regulation (EC) No 178/2002 and Regulation (EC) No 1223/2009 and Repealing Council Directives 90/385/EEC and 93/42/EEC (Text with EEA Relevance).

[53] Cioeta R., Cossu A., Giovagnoni E., Rigoni M., Muti P. (2022). A New Platform for Post-Marketing Surveillance and Real-World Evidence Data Collection for Substance-Based Medical Devices. Front. Drug Saf. Regul..

[54] Lundell S., Toots A., Sönnerfors P., Halvarsson A., Wadell K. (2022). Participatory Methods in a Digital Setting: Experiences from the Co-Creation of an eHealth Tool for People with Chronic Obstructive Pulmonary Disease. BMC Med. Inform. Decis. Mak..

[55] RAOSOFT Sample Size Calculator. http://www.raosoft.com/samplesize.html.

[56] Jaksa A., Arena P.J., Hanisch M., Marsico M. (2025). Use of Real-World Evidence in Health Technology Reassessments Across 6 Health Technology Assessment Agencies. Value Health.

[57] Hou M., Tang S., Zhang F., Fu S., Ding H., Cha Y., Ma X., Shi Y., Cai Y. (2025). Chemical Exposure in Females of Childbearing Age Associated with Sex Hormones: Evidence from an Untargeted Exposomic Approach. Environ. Int..

[58] Cioeta R., Muti P., Rigoni M., Morlando L., Siragusa F., Cossu A., Giovagnoni E. (2022). Effectiveness and Tolerability of Poliprotect, a Natural Mucosal Protective Agent for Gastroesophageal Reflux Disease and Dyspepsia: Surveys from Patients, Physicians, and Pharmacists. Front. Drug Saf. Regul..

[59] Fogacci F., ALGhasab N.S., Di Micoli V., Giovannini M., Cicero A.F.G. (2024). Cholesterol-Lowering Bioactive Foods and Nutraceuticals in Pediatrics: Clinical Evidence of Efficacy and Safety. Nutrients.

[60] Ghavami A., Ziaei R., Talebi S., Barghchi H., Nattagh-Eshtivani E., Moradi S., Rahbarinejad P., Mohammadi H., Ghasemi-Tehrani H., Marx W. (2023). Soluble Fiber Supplementation and Serum Lipid Profile: A Systematic Review and Dose-Response Meta-Analysis of Randomized Controlled Trials. Adv. Nutr..

[61] Vlachos D., Malisova S., Lindberg F.A., Karaniki G. (2020). Glycemic Index (GI) or Glycemic Load (GL) and Dietary Interventions for Optimizing Postprandial Hyperglycemia in Patients with T2 Diabetes: A Review. Nutrients.

[62] Alahmari L.A. (2024). Dietary Fiber Influence on Overall Health, with an Emphasis on CVD, Diabetes, Obesity, Colon Cancer, and Inflammation. Front. Nutr..

[63] Kim J., Baek Y., Lee S. (2024). Consumption of Dietary Fiber and APOA5 Genetic Variants in Metabolic Syndrome: Baseline Data from the Korean Medicine Daejeon Citizen Cohort Study. Nutr. Metab..

[64] Veluvali A., Snyder M. (2023). Dietary Fiber Deficiency in Individuals with Metabolic Syndrome: A Review. Curr. Opin. Clin. Nutr. Metab. Care.

[65] Greco C.M., Garetto S., Montellier E., Liu Y., Chen S., Baldi P., Sassone-Corsi P., Lucci J. (2020). A Non-Pharmacological Therapeutic Approach in the Gut Triggers Distal Metabolic Rewiring Capable of Ameliorating Diet-Induced Dysfunctions Encompassed by Metabolic Syndrome. Sci. Rep..

[66] McRorie J.W. (2015). Evidence-Based Approach to Fiber Supplements and Clinically Meaningful Health Benefits, Part 1: What to Look for and How to Recommend an Effective Fiber Therapy. Nutr. Today.

[67] Guarino G., Della Corte T., Strollo F., Gentile S. (2021). Policaptil Gel Retard in Adult Subjects with the Metabolic Syndrome: Efficacy, Safety, and Tolerability Compared to Metformin. Diabetes Metab. Syndr. Clin. Res. Rev..

[68] Holscher H.D. (2017). Dietary Fiber and Prebiotics and the Gastrointestinal Microbiota. Gut Microbes.

[69] Proietti G., Burico M., Quintiero C.M., Giovagnoni E., Mercati V., Gianni M., Mattoli L. (2023). Ready Biodegradability Study and Insights with Ultra-high-performance Liquid Chromatograph Coupled to a Quadrupole Time of Flight of a Metformin-based Drug and of Metarecod, a Natural Substance-Based Medical Device. J. Mass Spectrom..

