# Supplementary material for: From Altered Metabolic and Anthropometric Parameters to Metabolic Syndrome: A Cross-Sectional Survey on the Effectiveness and Safety of Neo-Policaptil® Gel Retard
Source: Healthcare (Basel). 2025 Dec 15;13(24):3293. doi: 10.3390/healthcare13243293 (PMC12732610; doi:10.3390/healthcare13243293)
Supplement: Supplementary file 1 [file healthcare-13-03293-s001.zip › healthcare-3971937-supplementary_revision.pdf]

Questionnaires

a. Patients and Child Caregivers

0_Age (single choice)

Answer
14-16 years
17-30 years
31-50 years
51-64 years
=65 years

0_Gender (single choice)

Answer
Female
Male

0_Instruction Level (single choice)

Answer
Obligatory schooling
High school diploma or technical/vocational institute
Bachelor’s, Master’s, Doctorate’s degree
Other

0_Birth Region (single choice)

Answer
Abruzzo
Basilicata
Calabria
Campania
Emilia-Romagna
Friuli-Venezia Giulia
Lazio
Liguria
Lombardy
Marche
Molise
Piedmont
Apulia
Sardinia
Sicily
Tuscany
Trentino-South Tyrol
Umbria
Aosta Valley
Veneto
Other

0_Residence Region (single choice)

Answer
Abruzzo
Basilicata
Calabria
Campania
Emilia-Romagna
Friuli-Venezia Giulia
Lazio
Liguria
Lombardy
Marche
Molise
Piedmont
Apulia
Sardinia
Sicily
Tuscany
Trentino-South Tyrol
Umbria
Aosta Valley
Veneto
Other

1_Have you had any experience of using Metarecod granular sachets in the past year? (single choice)

Answer
Yes, as a person who has used the product
Yes, as the parent/guardian who gave the product to a child
No, I have not yet used the product

Consumers/patients results on child administration

2_Where do you usually purchase the product? (multiple choice)

Answer
Pharmacy
Parapharmacy
Internet
Herbalist shop
Other (e.g. large-scale retail channels)

3_The child to whom you last administered Metarecod granular sachets is: (single choice)

Answer
Male
Female

4_How old is the child you last administered Metarecod granular sachets to? (single choice)

Answer
From 8 to 10 years
From 11 to 12 years of age
For over 12 years
Other

5_Do you usually tend to find a solution to your children's symptoms without consulting a doctor? (self-medication) (single choice)

Answer
No
Yes

6_Which of the following conditions do you use Metarecod granular sachets for in a child? (multiple choice)

Answer
Cholesterol (in case of LDL* =100 mg/dl or HDL cholesterol** < 40 mg/dl in males and < 50 mg/dl in females)
Triglycerides (in case of values =150 mg/dl)
Blood sugar (in case of fasting values >100 mg/dl (IFG) or impaired tolerance or reduced glucose tolerance 140-199 mg/dl (IGT))
High values of abdominal circumference for even in normal weight subjects
Metabolic syndrome diagnosed by a doctor
None of the above

8_What are the main altered parameters, apart from the high abdominal girth, that characterise the child's medically diagnosed metabolic syndrome? (multiple choice)

Answer
Cholesterol (HDL cholesterol < 40 mg/dl in males and < 50 mg/dl in females)
Triglycerides (in case of values = 150 mg/dl)
Blood sugar (in case of fasting values >100 mg/dl (IFG) or impaired tolerance or reduced glucose tolerance 140-199 mg/dl (IGT)
High blood pressure
I don't know

9_To what extent has the child's condition improved as a result of using Metarecod granular sachets (single choice)

Answer
Excellent
Good
Fair
Adequate
Poor

10_Please indicate how satisfied you are with the efficacy of Metarecod granular sachets (single choice)

Answer
Excellent
Good
Fair
Adequate
Poor

11_What was the duration of the child's single course of treatment with Metarecod granular sachets? (single choice)

Answer
1 month
2 months
3 months
I did not finish yet the first month of treatment
Other

13_How many granular sachets of Metarecod granular sachets do you generally give your child? (single choice)

Answer
One sachet per day
Two sachets per day
Other

14_Do you usually give your child Metarecod granular sachets before the two main meals? (single choice)

Answer
No
Yes

15_Do you usually have the beverage made with Metarecod granular sachets taken by the child before it gives rise to gel formation in the glass? (single choice)

Answer
Yes
No
I don’t know

16_Do you usually make your child drink an additional half glass of water after taking Metarecod granular sachets? (single choice)

Answer
No
Yes

17_How long after being treated with Metarecod granular sachets did you notice the first beneficial effects in your child? (single choice)

Answer
In less than 1 month
In 1 month
In 2 months
In 3 months
In more than 3 months
I haven’t seen any improvements

18_Do you use anything other than Metarecod granular sachets for your child’s problem? (multiple choice)

Answer
No, he only takes Metarecod granular sachets
Diet therapy
Physical activity
Metformin
Liraglutide
Statins
Other

19_Do you usually have your child take Metarecod granular sachets two hours apart from other medications? (single choice)

Answer
Yes
No
The child generally does not take other medications

20_How much did the child’s quality of life (mood, nutrition, physical activity, ability to play, social life, tiredness, sleep, attention) improve with Metarecod granular sachets? (single choice)

Answer
Extremely
Greatly
Moderately
Somewhat
Not at all

21_Indicate whether any of these conditions have occurred since the child took Metarecod granular sachets: (multiple choice)

Answer
Her/His mood has improved
She/He can eat more freely
Her/His physical activity improved
Her/His ability to play has improved
Her/His social life has improved
Her/His tiredness has decreased
Her/His sleep has improved
Her/His attention in daily actions has improved

22_To what extent has your quality of life, as a carer, improved with the use of Metarecod granular sachets? (single choice)

Answer
Extremely
Greatly
Moderately
Somewhat
Not at all

Consumers/patients results on direct use of the product

23_Do you usually tend to find a solution to your symptoms without consulting a doctor? (self-medication) (single choice)

Answer
No
Yes

24_Where do you usually purchase the product? (multiple choice)

Answer
Pharmacy
Parapharmacy
Internet
Herbalist shop
Other (e.g. large-scale retail channels)

25_For which of the following conditions do you usually use Metarecod granular sachets? (multiple choice)

Answer
Cholesterol (in case of LDL* =100 mg/dl or HDL cholesterol** < 40 mg/dl in males and < 50 mg/dl in females)
Triglycerides (in case of values = 150 mg/dl)
Blood sugar (in case of fasting values >100 mg/dl (IFG) or impaired or reduced glucose tolerance 140-199 mg/dl (IGT),
High values of abdominal circumference even in normal weight subjects (= 94 cm in males and = 80 cm in females)
Metabolic syndrome diagnosed by a doctor
None of the above

27_What are the main altered parameters, apart from high abdominal circumference, that characterize your doctor-diagnosed metabolic syndrome? (multiple choice)

Answer
Cholesterol (HDL cholesterol < 40 mg/dl in males and < 50 mg/dl in females)
Triglycerides (in case of values = 150 mg/dl)
Blood sugar (in case of fasting values >100 mg/dl (IFG) or impaired tolerance or reduced glucose tolerance 140-199 mg/dl (IGT)
High blood pressure
I don't know

28_To what extent has your condition improved with the use of Metarecod granular sachets? (single choice)

Answer
Extremely
Greatly
Moderately
Somewhat
Not at all

29_Please indicate how satisfied you are with the efficacy of Metarecod granular sachets (single choice)

Answer
Excellent
Good
Fair
Adequate
Poor

30_What was the duration of the single cycle of treatment with Metarecod granular sachets? (single choice)

Answer
1 month
2 months
3 months
I did not finish yet the first month of treatment
Other

32_How many Metarecod granular sachets do you generally take? (single choice)

Answer
One sachet per day
Two sachets per day
Other

33_Do you usually take Metarecod granular sachets before your main meals? (single choice)

Answer
No
Yes

34_Do you usually take the beverage obtained with Metarecod granular sachets before it forms a gel in the glass? (single choice)

Answer
Yes
No
I don't know

35_Do you usually drink an additional half glass of water after taking Metarecod granular sachets? (single choice)

Answer
No
Yes

36_How long after being treated with Metarecod granular sachets did you notice the first beneficial effects? (single choice)

Answer
In less than 1 month
In 1 month
In 2 months
In 3 months
In more than 3 months
I haven't seen any improvements

37_Do you use anything other than Metarecod granular sachets for your problem? (multiple choice)

Answer
No, I only take Metarecod granular sachets
Diet therapy
Physical activity
Metformin
Orlistat
Liraglutide
Statins
Other

38_Do you usually take Metarecod granular sachets two hours apart from other medications? (single choice)

Answer
Yes
No
I don't take other medications

39_To what extent has your quality of life (mood, freedom of nutrition, physical activity, social life, tiredness, concentration, performance at work) improved with Metarecod sachets granular? (single choice)

Answer
Extremely
Greatly
Moderately
Somewhat
Not at all

40_Please indicate if you have experienced any of these conditions since you have used Metarecod granular sachets (multiple choice)

Answer
My mood has improved
I can eat more freely
My performance at physical activity has improved
My social relations have improved
My performance at work has improved
My tiredness has decreased
My sleep has improved
My focus in daily actions has improved

41_How do you rate the safety and tolerability of the product Metarecod granular sachets? (single choice)

Answer
Excellent
Good
Fair
Adequate
Poor

42_How do you rate the clarity of the information contained within the box and on the informational leaflet for Metarecod granular sachets in terms of the "Indications"? (single choice)

Answer
Excellent
Good
Fair
Adequate
Poor

43_How do you rate the clarity of the information contained within the box and on the informational leaflet for Metarecod granular sachets in terms of the "Instructions for use"? (single choice)

Answer
Excellent
Good
Fair
Adequate
Poor

44_How do you rate the clarity of the information contained within the box and on the informational leaflet for Metarecod granular sachets in terms of the "Warnings"? (single choice)

Answer
Excellent
Good
Fair
Adequate
Poor

46_How were you introduced to the product? (multiple choice)

Answer
Recommended by the Doctor
Recommended by the Pharmacist
Recommended by herbalist shop
Recommended by an acquaintance
Via advertising
Via internet
Other

b. Pharmacist

1_Have you collected any feedback on your customers' use of Metarecod granular sachets in the last year? (single choice)

Answer
No
Yes

2_Please indicate the type of practice in which you dispense Metarecod granular sachets (single choice)

Answer
Independent private pharmacy
Pharmacy belonging to a private chain of pharmacies
Municipal pharmacy
Pharmacy specialised in natural products
Herbalist shop
Independent parapharmacy
Parapharmacy belonging to a private chain of parapharmacies
Mass retail parapharmacy

4_For what conditions do you recommend Metarecod granular sachets (multiple choice)

Answer
Cholesterol (in case of LDL* =100 mg/dl or HDL cholesterol** < 40 mg/dl in males and < 50 mg/dl in females)
Triglycerides (in case of values = 150 mg/dl)
Blood sugar (in case of fasting values ??>100 mg/dl (IFG) or impaired tolerance or reduced glucose tolerance 140-199 mg/dl (IGT))
People with high abdominal circumference values ??even in normal weight subjects (= 94 cm in males and = 80 cm in females)
People who present a clinical picture characteristic of metabolic syndrome, but without a diagnosis established by a doctor
Persons with a medically established diagnosis of Metabolic Syndrome
People with other types of disorders (Specify which ones)

5_How do you rate the overall effectiveness of Metarecod granular sachets (single choice)

Answer
Excellent
Good
Fair
Adequate
Poor

6_How satisfied are you with the improvements brought about Metarecod granular sachets on balancing the metabolic parameter “Cholesterol (in case of LDL* =100 mg/dl or HDL cholesterol < 40 mg/dl in males and < 50 mg/dl in females)” in your customers? (single choice)**

Answer
Extremely
Greatly
Moderately
Somewhat
Not at all
I don’t know

7_How satisfied are you with the improvements brought about Metarecod granular sachets on balancing the metabolic parameter “Triglycerides (in case of values = 150 mg/dl)” in your customers? (single choice)

Answer
Extremely
Greatly
Moderately
Somewhat
Not at all
I don’t know

8_How satisfied are you with the improvements brought about Metarecod granular sachets on balancing the metabolic parameter “Blood sugar (in case of fasting values >100 mg/dl (IFG) or impaired tolerance or reduced glucose tolerance 140-199 mg/dl (IGT)” in your customers? (single choice)

Answer
Extremely
Greatly
Moderately
Somewhat
Not at all
I don’t know

9_How satisfied are you with the improvements brought about Metarecod granular sachets in case of “high abdominal circumference values even in normal weight subjects (= 94 cm in males and = 80 cm in females)” in your customers? (single choice)

Answer
Extremely
Greatly
Moderately
Somewhat
Not at all
I don’t know

10_How satisfied are you with the improvements brought about by Metarecod granular sachets in the case of ‘Metabolic Syndrome’ in your customers? (single choice)

Answer
Extremely
Greatly
Moderately
Somewhat
Not at all
I don’t know

11_Please specify the most common age group who use Metarecod granular sachets (multiple choice)

Answer
8-11 years
12-17 years
18-30 years
31-50 years
51-64 years
=65 years

12_In general, what is the duration of treatment with Metarecod granular sachets in your customers? (multiple choice)

Answer
1 month
2 months
3 months
Other
I don’t know

13_Your customers usually repeat treatment with Metarecod granular sachets? (multiple choice)

Answer
Yes. They usually resume treatment within 1 month of stopping it
Yes. They usually resume treatment within 2 months of stopping it
Yes. They resume treatment after a very variable average time after the interruption
No. They change the treatment
I don’t know

14_Do you usually recommend taking Metarecod granular sachets before two main meals? (single choice)

Answer
No
Yes

15_How soon after starting treatment with Metarecod granular sachets have you been told by your clients that their clinical picture has improved? (multiple choice)

Answer
In less than 1 month
In 1 month
In two months
In 3 months
In more than 3 months
I didn't notice any improvements
I don't know

16_How would you assess the effect of treatment with Metarecod granular sachets on quality of life (mood, freedom of nutrition, physical activity, social life, tiredness, concentration, performance at work), as reported by your customers? (single choice)

Answer
Excellent
Good
Fair
Adequate
Poor
I don't know

17_Please could you describe generally how the customers to whom you dispense Metarecod granular sachets use the product? (multiple choice)

Answer
As the only therapy in those who have not yet used other therapies
As the only therapy in those who have used other therapies without success
In association with Diet therapy
In association with Physical activity
In association with Metformin
In association with Orlistat
In association with Liraglutide
In association with Statins
Other
I don't know

18_Do you usually recommend taking Metarecod granular sachets two hours apart from other medications? (single choice)

Answer
No
Yes

19_How would you evaluate the safety and tolerability of Metarecod granular sachets? (single choice)

Answer
Excellent
Good
Fair
Adequate
Poor

20_Have you experienced or reported any interactions between Metarecod granular sachets and other treatments your customers are receiving? (single choice)

Answer
No
Yes

21_How would you evaluate the clarity of the information contained on the carton and package leaflet of Metarecod granular sachets with regard to "Instructions for use"? (single choice)

Answer
Excellent
Good
Fair
Adequate
Poor

22_How would you evaluate the clarity of the information contained on the carton and package leaflet of Metarecod granular sachets with regard to "Warnings"? (single choice)

Answer
Excellent
Good
Fair
Adequate
Poor

23_How would you evaluate the clarity of the information contained on the carton and package leaflet of Metarecod granular sachets with regard to “Indications”? (single choice)

Answer
Excellent
Good
Fair
Adequate
Poor

24_From a scientific viewpoint how would you evaluate the quality of information you receive from different communication channels (sales agents, training tutor, scientific information reps, publications, website, etc.)? (single choice)

Answer
Excellent
Good
Fair
Adequate
Poor

c. Physicians

1_Have you had any prescribing experience with Metarecod granular sachets in the last year? (single choice)

Answer
No
Yes

2_Please state your profession (single choice)

Answer
General Practitioner
Gastroenterologist
Internist
Dietician
Nutritionist
Pediatrician
Endocrinologist
Other

4_For what conditions do you prescribe Metarecod granular sachets (multiple choice)

Answer
LDL cholesterol (= 100 mg/dl)
HDL cholesterol (< 40 mg/dl in males and < 50 mg/dl in females)
Triglycerides (in case of values = 150 mg/dl)
Blood sugar (in case of fasting values > 100 mg/dl (IFG) or impaired tolerance or reduced glucose tolerance 140-199 mg/dl (IGT))
High values of abdominal circumference even in normal weight subjects (= 94 cm in males and = 80 cm in females)
Metabolic syndrome
People with other types of disorders

5_How would you rate the overall effectiveness of Metarecod granular sachets? (single choice)

Answer
Excellent
Good
Fair
Adequate
Poor

6_How satisfied are you with the improvements brought about by Metarecod granular sachets on balancing the metabolic parameter 'LDL cholesterol (=100 mg/dl)' in your patients? (single choice)

Answer
Extremely
Greatly
Moderately
Somewhat
Not at all
I don't know

7_How satisfied are you with the improvements brought about by Metarecod granular sachets on the rebalancing of the metabolic parameter 'HDL cholesterol (< 40 mg/dl in males and < 50 mg/dl in females)' in your patients? (single choice)

Answer
Extremely
Greatly
Moderately
Somewhat
Not at all
I don't know

8_How satisfied are you with the improvements brought about by Metarecod granular sachets on the rebalancing of the metabolic parameter 'Triglycerides (in case of values =150 mg/dl)' in your patients? (single choice)

Answer
Extremely
Greatly
Moderately
Somewhat
Not at all
I don't know

9_How satisfied are you with the improvements brought about by Metarecod granular sachets on the rebalancing of the metabolic parameter 'Blood glucose (in case of fasting values >100 mg/dl (IFG) or impaired tolerance or impaired glucose tolerance 140-199 mg/dl (IGT)' in your patients? (single choice)

Answer
Extremely
Greatly
Moderately
Somewhat
Not at all
I don't know

10_How satisfied are you with the improvements brought about by Metarecod granular sachets in the case of 'High abdominal girth values even in normal-weight subjects = 94 cm in males and = 80 cm in females)' in your patients? (single choice)

Answer
Extremely
Greatly
Moderately
Somewhat
Not at all
I don't know

11_How satisfied are you with the use of Metarecod granular sachets for 'Metabolic Syndrome' in your patients? (single choice)

Answer
Extremely
Greatly
Moderately
Somewhat
Not at all
I don't know

12_Please specify the most common age group of patients (multiple choice)

Answer
8-11 years
12-17 years
18-30 years
31-50 years
51-64 years
=65 years

13_What is the general duration of treatment with Metarecod granular sachets among your patients? (multiple choice)

Answer
1 month
2 months
3 months
Other

14_Do your patients usually repeat treatment with Metarecod granular sachets? (multiple choice)

Answer
Yes. They usually resume treatment within 1 month of stopping it
Yes. They usually resume treatment within 2 months of stopping it
Yes. They resume treatment after a very variable average time after the interruption
No. They change the treatment
I don’t know

15_Do you usually recommend taking Metarecod granular sachets before two main meals? (single choice)

Answer
No
Yes

16_How soon after starting treatment with Metarecod granular sachets did you notice an improvement in the clinical picture in your patients? (multiple choice)

Answer
In less than 1 month
In 1 month
In two months
In 3 months
In more than 3 months
I didn’t notice any improvements
I don’t know

17_How do you assess the effect of treatment with Metarecod granular sachets on the quality of life (mood, freedom from food, physical activity, social life, work, tiredness, sleep, concentration) reported by your patients? (single choice)

Answer
Excellent
Good
Fair
Adequate
Poor
I don’t know

18_In general, how do you prescribe Metarecod granular sachets to your patients? (multiple choice)

Answer
As the only therapy in those who have not yet used other therapies
As the only therapy in those who have used other therapies without success
In association with Diet
In association with Physical activity
In association with Metformin
In association with Orlistat
In association with Liraglutide
In association with Statins
Other

19_Do you usually recommend taking Metarecod granular sachets two hours apart from other medications? (single choice)

Answer
Yes
No

20_How would you evaluate the safety and tolerability of Metarecod granular sachets? (single choice)

Answer
Excellent
Good
Fair
Adequate
Poor

21_Have you experienced any interactions between Metarecod granular sachets and any other treatments your patients are receiving? (single choice)

Answer
No
Yes

22_How would you evaluate the clarity of the information contained on the carton and package leaflet of Metarecod granular sachets with regard to “Warnings”? (single choice)

Answer
Excellent
Good
Fair
Adequate
Poor

23_How would you evaluate the clarity of the information contained on the carton and package leaflet of Metarecod granular sachets with regard to “Indications”? (single choice)

Answer
Excellent
Good
Fair
Adequate
Poor

24_How would you evaluate the clarity of the information contained on the carton and package leaflet of Metarecod granular sachets with regard to “Instructions for use”? (single choice)

Answer
Excellent
Good
Fair
Adequate
Poor

25_From a scientific viewpoint how would you evaluate the quality of information you receive from different communication channels (sales agents, training tutor, scientific information reps, publications, website, etc.)? (single choice)

Answer
Excellent
Good
Fair
Adequate
Poor
